# Birth intervals and breast cancer risk

**DOI:** 10.1038/sj.bjc.6605300

**Published:** 2009-09-08

**Authors:** A Kauppila, P Kyyrönen, M Hinkula, E Pukkala

**Affiliations:** 1 Department of Obstetrics and Gynecology, Oulu University Hospital, FI-90250 Oulu, Finland; 2 Finnish Cancer Registry, Institute for Statistical and Epidemiological Cancer Research, Pieni Roobertinkatu 9, FI-00130, Helsinki, Finland; 3 School of Public Health, FI-33014 University of Tampere, Tampere, Finland

**Keywords:** aetiology of breast cancer, risk factor, pregnancy, age at first birth, birth interval, interval from birth to cancer

## Abstract

**Background:**

The interval between successive births (birth interval) may affect breast cancer risk, whereas interval from last birth to cancer onset may modify its behaviour.

**Methods:**

The study cohort consisted of 29 488 Finnish grand multiparous (GM) women, including 628 women with breast cancer. Conditional logistic regression for case–control design nested within the cohort was used to estimate proportional hazards (referred as relative risks, RR). Age at first birth and parity were co-variables.

**Results:**

Short interval (<1 year) between first and second birth increased the risk of advanced ductal breast cancer at ages < 50 years (RR=5.29; 95% CI 2.00–14.0) as compared to interval 3+ years. The risk of advanced ductal cancer was also large (RR = 4.00; 95% CI 1.19–13.4) shortly (<3 years) after last birth as compared with the period 15+ years.

**Conclusions:**

Short birth interval-associated excess breast cancer risk may be related to stimulatory effects of female steroid hormones produced during two closely connected pregnancies, or defective breast maturation owing to failures in breastfeeding.

Findings in our previous study of grand multiparous women (GM, at least five births) indicated that birth interval (time between two successive births) might affect the risk of breast cancer (Hinkula *et al*, 2001). This finding and those of others (Albrektsen *et al*, 2005) prompted us to evaluate the impact of individual birth intervals on risk. Specific attention was paid to the interval between first and second births because the first pregnancy had a significant role in the maturation of the breast resistance against carcinogenic influences (Russo *et al*, 1994a, 2005). The length of birth interval depends also on the duration of breastfeeding, which is an important determinant of breast cancer risk (Collaborative Group of Hormonal in Breast Cancer, 2002; Ursin *et al*, 2005; Lord *et al*, 2008).

Pregnancies have dual effects on breast cancer: an immediate increase in the risk after childbirth is followed by a long-term protection (Woods *et al*, 1980; Kvåle and Heuch, 1987; Kelsey *et al*, 1993). In addition, cancers appearing within 2–6 years after birth are more frequently advanced than those in women having a long interval between birth and cancer onset (Kroman *et al*, 1997; Wohlfarth *et al*, 2001; Phillips *et al*, 2004; Rosenberg *et al*, 2004; Albrektsen *et al*, 2005, 2006).

This study population comprises of only GM women, most of whom belong to the religious minority, the Laestadian movement within the Lutheran church, which forbids the use of artificial contraception. Hence conception in this population takes place in physiological circumstances, thereby representing a specific advantage for our study. Here, we aimed to explore the impact of individual birth intervals on breast cancer risk at different stages. Another aim was to assess how the relative risk changes in relation to time since last birth in a Finnish cohort of GM women.

## Materials and methods

The data of the Finnish national Population Register comprised 29 488 women born 1935 or later and registered as having at least five biological children by the end of 1997. This database involves complete linkages between parents and the children born in October 1953 or later, but precluding our obtaining full parity history for women born before 1935. The exactly registered birthdays of each child form the basis for birth interval calculations.

Breast cancer cases diagnosed among the GM women between the fifth childbirth and 31 December 2006 were identified in an automatic record linkage with the files of the national population-based Finnish Cancer Registry, using personal identifiers as the key. There were 628 such breast cancers. The Finnish Cancer Registry files include clinical stage and cancer morphology. The registry receives cancer notifications from hospitals, pathological and cytological laboratories, and also from physicians outside hospitals; its coverage is almost 100% (Teppo *et al*, 1994).

### Statistical methods

For each breast cancer case, 50 controls were randomly selected among the GM cohort members who were at risk for breast cancer at the time of cancer onset of the case and fulfilled the matching criteria; a tolerance of ±1 year was allowed on the date of birth. The proportional hazards method was applied to this case–control data. A woman was a non-case until she became a case, so controls for each case were selected among non-cases irrespective of whether they later became cases. The point estimates, hazard ratios were obtained in SAS 9.1 using the PHREG procedure with the option TIES=DISRETE, which requests the discrete logistic model. This method is same as the method of the conditional logistic regression analysis if the controls were selected among the individuals who were at risk of becoming cases. Proportional hazard were referred here as relative risks (RR). Possible interactions were evaluated using the TPHREG procedure, a test release of the PHREG procedure that incorporates the CLASS statement in SAS 9.1. No interactions were found between the study variables.

The RRs were also calculated for ductal and lobular, and for local and advanced (regional or distant metastases) breast cancers separately. Further stratification was based on the age at the diagnosis of cancer (<50 years and 50+ years). Pregnancy at the age of 50 years or later is extremely rare. The younger women represent thus the years of reproduction and the older ones that of ovarian quiescence. The distribution of patients into different subcategories is presented in Table 1.

The likelihood ratio test was used to evaluate statistical significance of the parameters of interest. The trend test was analysed using linear trend test for the classified variables. The lowest category had value 1 and the highest category had the same value as the number of classes of the respective variable. Each of the four intervals between first and fifth births was classified into three categories (<1, 1–2 and 3+ complete years). Parity (5, 6, 7, 8, 9 and 10+ children) and the age at first birth (<20, 20–24, 25–29, 30+ years) were added in each model. The time from the latest birth to the date of onset of cancer was stratified into four categories (<3, 3–6, 7–14, 15+ complete years).

## Results

Overall, the RR did not differ significantly between different birth intervals, whereas short interval (<3 years) between last birth and cancer onset was associated with a significantly increased risk as compared to those with the interval of 15+ years (Table 2). Increase in the number of births and decline in age at first birth diminished the risk of breast cancer.

For cancers diagnosed before 50 years of age, the RR for short interval (<1 year) between first and second birth was increased twofold when compared with birth interval of 3+ years, and the interval from last birth to cancer onset among the cases was three times more often short (<3 years) than among the controls (Table 3). An increase in parity from 5 to 8+ nearly halved the RR in both age groups. In the group of 50+ year-old GM women, a decline in age at first birth decreased significantly the RR of breast cancer.

The most significant findings appeared in advanced ductal cancer diagnosed before age 50 years (Table 4). Short (<1 year) interval between first and second birth was associated with more than fivefold increased RR when compared with the 3+ years category. The RR of this cancer type appearing within 3 years after last birth was fourfold as compared with the 15+ years category. These factors operate separately; there was not a single breast cancer GM woman, with birth interval <1 year and interval from last birth to cancer <3 years. A long interval (15+ years) between last birth and cancer onset may protect against breast cancer at age 50+ years (Table 4).

## Discussion

This study finds that among GM women, breast cancer is associated with birth interval. Most important was the new finding that short birth interval between first and second birth was significantly associated with increased risk of advanced ductal cancer in young GM mothers. The risk of ductal breast cancer in clinically advanced stage is also high during the 3 years following the last birth.

Previous studies on the risk factors in breast cancer have comprised predominantly nulliparous women and women with few children. We widened the perspective by evaluating this topic in a homogenous group of Finnish GM women, whose breast cancer risk is low, 45% having below the average incidence (Hinkula *et al*, 2001).

The registers used in this study, the National Population Register for births, and the Finnish Cancer Registry, are reliable and virtually complete (Teppo *et al*, 1994; Pukkala, 2009). Because the most important results in this study are from ages <50 years, the lack of information on postmenopausal hormone therapy or body weight does not weaken the validity of the findings.

The RR of advanced ductal breast cancer of GM women below 50 years of age was more than five times higher if the interval between first and second birth was <1 year (=pregnancy interval <3–4 months) as compared to 3+ years. The mechanism of this detrimental effect is unclear. The fact that it appeared only after the first pregnancy might reflect an association with breast maturation, because first pregnancy may have a central role in the differentiation of breast cells more resistant to carcinogenic influences (Lambe *et al*, 1994; Russo and Russo, 1994b; Chie *et al*, 2000; Russo *et al*, 2005; Wagner and Smith, 2005; Siwko *et al*, 2008). The suggestion that incomplete differentiation may predispose to breast cancer (Lagiou *et al*, 2003) has been supported by findings in experimental animal studies that an interaction of carcinogen with an undifferentiated breast epithelium is a prerequisite for carcinogenic initiation (Russo *et al*, 2001). It is possible that an insufficiently maturated breast of the young mother after her first childbirth – remains susceptible to carcinogenic effects, including hormonal influences.

Theories of hormonal mechanisms of breast cancer development are primarily based on exposure to excessive endogenous oestrogens (Colditz and Rosner, 2000; Yager and Davidson 2006). The influence of endogenous hormones on the risk of breast cancer of women may involve in addition to estrogens effects of progesterone, prolactin, testosterone and IGF-1 on terminal differentiation of mammary stem cells (Merrill *et al*, 2005). Estrogens and progesterone are mitogenic for epithelial breast cells, and may stimulate breast cell proliferation (Söderqvist, 1998). During pregnancy, progesterone induces lobular–alveolar development and differentiation for lactation (Lange *et al*, 1999), and estrogens regulate ductal growth (Mauvais-Jarvis *et al*, 1986). Older premenopausal and perimenopausal women have been claimed to be at increased risk (Alexander and Roberts, 1987), possibly implying an increased growth rate for present tumours during the menopause, perhaps due to the effects of oestrogen unopposed by progesterone.

Studies on the possible role of prolactin in breast cancer have yielded inconsistent results – protective or carcinogenic effects or no effect (Clevenger *et al*, 2003; Albrektsen *et al*, 2006). The most recent prospective study suggests that prolactin might increase the risk of breast cancer (Tworoger *et al*, 2007). During pregnancy, prolactin level rises strongly from the eighth week onwards reaching the peak concentration at term. Thereafter prolactin, the principal hormone for milk biosynthesis, remains elevated only in nursing mothers, in whom each breastfeeding induces a transient peak in its level. In non-lactating woman prolactin concentration declines rapidly, which allows early resumption of ovulatory menstrual cycle and early fecundation after the birth. The contraceptive effect of prolactin is not absolute; the efficacy weakens especially from the fourth postpartum month onward. Fecundation of lactating GM mother within 3–4 months after her first childbirth is thus possible but supposedly a rare phenomenon. In such a case, the joint actions of prolactin, (induced by suckling) and progesterone (from corpus luteum of the second pregnancy) would initiate, in the beginning of second pregnancy, abnormal cellular changes owing to the potential for breast cancer development. Alternatively, the long-term joint stimulatory actions of placental hormones (estrogens and progesterone) and prolactin during two closely consecutive pregnancies may serve as initiators for malignant transformation of epithelial breast cells.

In spite of our lack of breastfeeding data, we hypothesise that GM women, with increased breast cancer risk in association with a short interval between first and second birth, do not have breastfeed or have only for a very short time. Short-term breastfeeding, and especially no breastfeeding is associated with shorter birth interval (Rutstein 2005). An increase in breast cancer risk has been reported among premenopausal American women, who nursed their first (RR=1.37) or second (RR=1,44) child <1 month (Byers *et al*, 1985).

Because lactation participates in the differentiation of mammary epithelium in its terminal phase (Russo and Russo, 1994b; Russo *et al*, 2001, 2008), deficient breastfeeding of the first child might leave the breast cells susceptible to carcinogenic influences. This would be in accordance with the finding that lactation has a significant independent protective effect in breast cancer (Byers *et al*, 1985; Collaborative Group of Hormonal Factors in Breast Cancer, 2002; Ursin *et al*, 2005), particularly before menopause (Byers *et al*, 1985; Newcomb *et al*, 1994; Lord *et al*, 2008). Insufficient breastfeeding resulting in defective breast maturation might thus be the primary cause for breast cancer of young GM women in this specific subgroup.

The picture of breast cancer risk increase associated with birth intervals appeared to be more complicated than the previous one that long birth interval solely would affect the risk (Kvåle *et al*, 1987; Albrektsen *et al*, 2005, 2006). In the light of the present results the picture of adverse effects associated with birth intervals seems to be more complex than the view that only long birth intervals affect risk (Kvåle and Heuch, 1987; Albrektsen *et al*, 2005, 2006).

The RR of breast cancer of young GM women was highest shortly after their last birth, which is in accordance with earlier observations of mothers with few pregnancies (Kvåle and Heuch, 1987; Leon *et al*, 1995; Wohlfarth *et al*, 2001; Albrektsen *et al*, 2005, 2006). Transiently increased breast cancer risk after the last childbirth in this study showed no association with the number of pregnancies. The detrimental effect of short interval extended to GM women aged 50+ years. Transient increase risk after latest birth may even last for 15 years (Lambe *et al*, 1994; Liu *et al*, 2002).

Young age at first birth and increasing parity are established protective factors in breast cancer risk (La Vecchia *et al*, 1989; Layde *et al*, 1989; Lambe *et al*, 1996; Merrill *et al*, 2005). In this study, after adjusting for other study variables, age at first birth and parity were significant factors in GM women aged 50+ years. Among GM women <50 years, our previous study of the same cohort (Hinkula *et al*, 2001) that did not include birth intervals and age at last birth showed, that age at first birth – but not parity – is a significant risk factor. In contrast, in this study parity is a significant risk factor among younger GM women – but not age at first birth. Thus, the new age-related variables seem to weaken the age at first birth effect and strengthen the role of parity as an independent risk factor in this age period.

Elevated risk of advanced ductal breast cancer of young GM women with short interval between first and second birth might have an association with female steroid hormones and prolactin secreted during two closely consecutive pregnancies or with defective maturation of the breast owing to inappropriate nursing.

## Figures and Tables

**Table 1:** Number (*N*) and percentage of women with breast cancer in 1974–2006 among 29 488 women in Finland born 1935+ and registered to have at least five biological children, by histology, clinical stage at diagnosis, and age at diagnosis

	**All ages**		**Age <50 years**		**Age 50+ years**	
**Breast cancer category**	**N**	**Percentage of all women**	**N**	**Percentage of sub-category**	**N**	**Percentage of sub-category**
All	628	100	180	29	448	71
*Histology*						
Ductal cancer	484	77	140	29	344	71
Lobular cancer	101	16	21	21	80	80
Other types/unknown	43	7	19	44	24	56
*Clinical stage*						
Local	326	52	67	21	259	80
Advanced	264	42	105	40	159	60
Unknown	38	6	8	21	30	79

**Table 2:** Number of breast cancer cases (*N*) among grand multiparous women, and model-based multivariable relative risks (RR) with 95% confidence intervals (95% CI), by study variables

**Variable**	**N**	**RR**	**95% CI**
*Interval between births*			
*1st and 2nd*			
<1 year	60	1.06	0.76–1.49
1–2.99 years	472	0.91	0.72–1.14
3+years	96	1.00	Ref.
			
*2nd and 3rd*			
<1 year	25	0.78	0.50–1.21
1–2.99 years	449	1.05	0.86–1.27
3+ years	154	1.00	Ref.
			
*3rd and 4th*			
<1 year	18	1.08	0.66–1.78
1–2.99 years	397	1.06	0.89–1.27
3+ years	213	1.00	Ref.
			
*4th and 5th*			
<1 year	12	0.91	0.51–1.64
1–2.99 years	326	1.03	0.86–1.22
3+ years	290	1.00	Ref.
			
*Time since last birth* a			
<3 years	32	2.36	1.31–4.27
3–6.99 years	45	1.54	0.98–2.41
7–14.99 years	164	1.25	0.96–1.63
15+ years	387	1.00	Ref.
			
*Number of births* b			
5	407	1.00	Ref.
6	119	0.79	0.63–0.97
7	41	0.66	0.47–0.93
8+	61	0.58	0.42–0.80
			
*Age at first birth* c			
<20 years	212	1.00	Ref.
20–24 years	303	0.97	0.81–1.16
25–29 years	95	1.45	1.12–1.89
30+ years	18	1.47	0.88–2.46

a Likelihood ratio test *P*=0.04, *P*-trend=0.006.

b Likelihood ratio test *P*=0.001, *P*-trend=0.0001.

c Likelihood ratio test *P*=0.01, *P*-trend=0.02.

**Table 3:** Number of breast cancer cases (*N*) and model-based relative risks (RR) with 95% confidence interval (95% CI) in <50 and 50+ year-old grand multiparous women, by age at follow-up and by study variables

	**Age at follow-up <50 years**			**Age at follow-up 50+ years**		
**Variable**	**N**	**RR**	**95% CI**	**N**	**RR**	**95% CI**
*Interval between births*						
*1st and 2nd birth*						
<1 year	20	2.03	1.08–3.83	40	0.81	0.54–1.21
1–2.99 years	136	1.29	0.82–2.03	336	0.80	0.61–1.04
3+ years	24	1.00	Ref^a^	72	1.00	Ref
						
*2nd and 3rd birth*						
<1 year	5	0.71	0.28–1.85	20	0.80	0.49–1.31
1–2.99 years	129	1.23	0.85–1.78	320	0.98	0.78–1.24
3+ years	46	1.00	Ref	108	1.00	Ref
						
*3rd and 4th birth*						
<1 year	3	0.85	0.26–2.80	15	1.14	0.66–1.96
1–2.99 years	116	1.26	0.89–1.78	281	1.00	0.81–1.24
3+ years	61	1.00	Ref	152	1.00	Ref
						
*4th and 5th birth*						
<1 year	6	1.68	0.70–4.03	6	0.64	0.28–1.45
1–2.99 years	86	0.86	0.61–1.23	240	1.12	0.91–1.37
3+ years	88	1.00	Ref	202	1.00	Ref
						
*Time since last birth*						
<3 years	31	3.27	1.42–7.51	1	13.8	1.32–144
3–6.99 years	40	2.13	1.07–4.26	5	1.50	0.58–3.90
7–14.99 years	86	1.75	1.00–3.05	78	1.10	0.80–1.51
15+years	23	1.00	Ref^b^	364	1.00	Ref
						
*Number of births*						
5	110	1.00	Ref^c^	297	1.00	Ref^d^
6	37	0.90	0.60–1.34	82	0.73	0.57–0.94
7	12	0.63	0.33–1.21	29	0.66	0.45–0.99
8+	21	0.55	0.29–1.02	40	0.59	0.40–0.87
						
*Age at first birth*						
<20 years	53	1.00	Ref	159	1.00	Ref^e^
20–24 years	92	1.04	0.73–1.49	211	0.93	0.75–1.15
25–29 years	29	1.22	0.73–2.04	66	1.52	1.12–2.06
30+ years	6	1.41	0.54–3.64	12	1.51	0.82–2.81
Total	180			448		

a *P-*trend =0.04.

b LR test *P*=0.05, *P-*trend=0.008.

c *P-*trend=0.04.

d LR test *P*=0.006, *P-*trend=0.001.

e LR test *P*=0.009, *P-*trend=0.04.

**Table 4:** Number of ductal breast cancer with regional or distant metastases at diagnosis (*N*), and model-based relative risks (RR) with 95% confidence interval (95% CI) in <50 and 50+ year-old grand multiparous women, by age at follow-up and study variables

	**Age <50 years**			**Age 50+ years**		
**Variable**	***N***	**RR**	**95% CI**	***N***	**RR**	**95% CI**
*Interval between births*						
*1st and 2nd birth*						
<1 year	13	5.29	2.00–14.0	13	0.80	0.39–1.65
1–2.99 years	60	2.08	0.93–4.68	78	0.57	0.35–0.92
3+ years	7	1.00	Ref^a^	24	1.00	Ref
						
*2nd and 3rd birth*						
<1 year	1	0.26	0.03–1.99	7	0.91	0.39–2.11
1–2.99 years	57	1.11	0.64–1.93	73	0.72	0.47–1.11
3+ years	22	1.00	Ref	35	1.00	Ref
						
*3rd and 4th birth*						
<1 year	2	1.13	0.25–5.01	2	0.68	0.16–2.88
1–2.99 years	50	1.16	0.68–1.96	77	1.19	0.79–1.82
3+ years	28	1.00	Ref	36	1.00	Ref
						
*4th and 5th birth*						
<1 year	2	1.38	0.31–6.23	4	1.49	0.52–4.27
1–2.99 years	45	1.26	0.75–2.11	56	1.03	0.69–1.54
3+ years	33	1.00	Ref	55	1.00	Ref
						
*Time since last birth*						
<3 years	16	4.00	1.19–13.4	1	—	—
3–6.99 years	19	2.43	0.85–6.93	1	2.21	0.25–20.0
7–14.99 years	36	1.84	0.78–4.37	19	1.61	0.84–3.09
15+ years	9	1.00	Ref^b^	94	1.00	Ref^c^
						
*Number of births*						
5	46	1.00	Ref	78	1.00	Ref^d^
6	17	0.88	0.48–1.61	30	0.98	0.62–1.54
7	6	0.65	0.26–1.64	2	0.15	0.04–0.63
8+	11	0.53	0.22–1.32	5	0.32	0.12–0.87
						
*Age at first birth*						
<20 years	24	1.00	Ref	50	1.00	Ref^e^
20–24 years	40	0.91	0.53–1.56	45	0.63	0.42–0.95
25–29 years	14	1.06	0.50–2.28	14	1.10	0.58–2.08
30+ years	2	0.76	0.16–3.67	6	2.51	0.97–6.48
						
Total	80			115		

a LR test *P*=0.003; *P*-trend=0.006.

b *P*-trend=0.03.

c *P*-trend=0.04.

d LR test *P*=0.0006; *P*-trend=0.003.

e LR test *P*=0.01.
